# Circular RNA circBCBM1 promotes breast cancer brain metastasis by modulating miR-125a/BRD4 axis

**DOI:** 10.7150/ijbs.58916

**Published:** 2021-07-22

**Authors:** Bo Fu, Wei Liu, Cui Zhu, Peng Li, Li Wang, Li Pan, Ke Li, Peiying Cai, Min Meng, Yiting Wang, Anqi Zhang, Wenqiang Tang, Meng An

**Affiliations:** 1 Department of Central Laboratory, Liaocheng People's Hospital, Medical College of Liaocheng University, Liaocheng, P.R. China; 2 Department of Neurology, Dongchang Fu People's Hospital, Liaocheng, P.R. China; 3 Department of Clinical Laboratory, Liaocheng People's Hospital, Medical College of Liaocheng University, Liaocheng, P.R. China; 4 Medical College of Liaocheng University, Liaocheng, P.R. China

**Keywords:** breast cancer brain metastasis, circBCBM1, miR-125a, BRD4, biomarker, therapeutic target

## Abstract

Circular RNAs (circRNAs) play critical roles in tumorigenesis and the progression of various cancers. We previously identified a novel upregulated circRNA, circBCBM1 (hsa_circ_0001944), in the context of breast cancer brain metastasis. However, the potential biological function and molecular mechanism of circBCBM1 in breast cancer brain metastasis remain largely unknown. In this study, we confirmed that circBCBM1 was a stable and cytoplasmic circRNA. Functionally, circBCBM1 promoted the proliferation and migration of 231-BR cells *in vitro* and growth and brain metastasis *in vivo*. Mechanistically, circBCBM1 acted as an endogenous miR-125a sponge to inhibit miR-125a activity, resulting in the upregulation of BRD4 (bromodomain containing 4) and subsequent upregulation of MMP9 (matrix metallopeptidase 9) through Sonic hedgehog (SHH) signaling pathway. Importantly, circBCBM1 was markedly upregulated in the breast cancer brain metastasis cells and clinical tissue and plasma samples; besides, circBCBM1 overexpression in primary cancerous tissues was associated with shorter brain metastasis-free survival (BMFS) of breast cancer patients. These findings indicate that circBCBM1 is involved in breast cancer brain metastasis via circBCBM1/miR-125a/BRD4 axis. CircBCBM1 may serve as a novel diagnostic and prognostic biomarker and potential therapeutic target for breast cancer brain metastasis.

## Introduction

Brain metastases represent the most common intracranial neoplasm in adults, and breast cancer is the second most common cause of brain metastases 1, 2. Approximately 10% to 30% of breast cancer patients will develop brain metastases 3. Breast cancer brain metastasis has become an increasingly common incidence as the improvement of systemic therapy and imaging surveillance 4. Breast cancer brain metastases often induce neurological impairments by affecting cognitive and sensory functions, and confer a poor prognosis with an approximately 80% mortality within one year after diagnosis 5. The initiation and progression of brain metastases are staged into primary tumor cells' invasion, intravasation, dissemination, extravasation and colonization 6. However, the biological mechanism underlying establishment and progression of breast cancer brain metastasis remains largely unknown. Identification of the cellular and molecular mechanisms underlying breast cancer brain metastasis is desperately needed to provide a basis for the development of innovative diagnostic biomarkers and therapeutic targets.

Circular RNAs (circRNAs) are an emerging subgroup of endogenous noncoding RNAs. CircRNAs are single-stranded, covalently closed RNA molecules that are generated by back-splicing of precursor mRNAs 7. CircRNAs are characterized by a stable loop structure, evolutionary conservation, and high cell type-, tissue- or developmental stage-specific expression 8. Compared with linear counterparts, circRNAs are more stable because they lack accessible ends and are thus resistant to exonuclease-mediated digestion 9. CircRNAs' consentaneous general functions include sequestration of microRNAs (miRNAs) or proteins, modulation of RNA polymerase II (Pol II) transcription and interference with pre-mRNA splicing, and even translation, while the exact biological function of most circRNAs remains largely unexplored 10. Aberrant circRNA levels were found implicated in various cancers, including hepatocellular carcinoma 11, 12, gastric cancer 13, oral squamous cell carcinoma 14, lung adenocarcinoma 15, colorectal cancer 16, etc.

In our previous study, we primarily revealed the circRNA profile related to breast cancer brain metastases and identified 406 differentially expressed circRNAs between the brain metastatic 231-BR cells and the parental nonspecific metastatic MDA-MB-231 cells 17. Among these circRNAs, has_circ_0001944 (termed as circBCBM1 for brief) was one of the most significantly upregulated molecules in breast cancer brain metastases. As a novel circRNA, its biological function and molecular mechanism in breast cancer brain metastasis await elucidation. In this study, we found circBCBM1 dramatically promoted the proliferation and migration of 231-BR cells *in vitro* and growth and brain metastasis *in vivo*. Further study revealed that circBCBM1 could function as a sponge of has-miR-125a-5p (abbreviated as miR-125a) to upregulate bromodomain containing 4 (BRD4) and then upregulate matrix metallopeptidase 9 (MMP9) through Sonic hedgehog (SHH) signaling pathway. Moreover, circBCBM1 was upregulated in the *in vitro*-cultured breast cancer brain metastasis cells and clinical tissue and plasma samples, and the overexpression of circBCBM1 in primary cancerous tissues was correlated with shorter brain metastasis-free survival (BMFS) of breast cancer patients. Therefore, circBCBM1 may act as an oncogene to promote breast cancer brain metastasis and may serve as a potential diagnostic and prognostic biomarker and therapeutic target for breast cancer brain metastasis.

## Materials and methods

### Clinical samples

In this research, we collected 13 pairs of breast cancer (BC) and adjacent normal breast tissues (NBT) (cohort 1), 6 breast cancer brain metastasis (BCBM) tissues (cohort 2), 20 BC and 20 BCBM patients' plasma samples (cohort 3), and 53 BCBM patients' primary tumor tissues (cohort 4) from Liaocheng People's Hospital (Liaocheng, China). CircBCBM1 expression at the tissue level was quantified in cohort 1 and 2, that at the plasma level was quantified in cohort 3, and Kaplan-Meier analysis of the correlation between circBCBM1 expression and BMFS was conducted with data of cohort 4. Specimens were identified by two pathologists independently. Clinical information of the enrolled patients was collected from their electronic medical records. Informed consents were obtained from all participants. The study was approved by the Ethics Committee of Liaocheng People's Hospital.

### Cell culture

The origin and culture conditions of human brain-targeting breast carcinoma cell line 231-BR and its parental cell line MDA-MB-231 were as described previously 17, 18. Breast cancer cell line BT-474 and T47D were purchased from American Type Culture Collection (ATCC; Manassas, VA, USA) and cultured in Dulbecco's Modified Eagle Medium (DMEM; Invitrogen, CA, USA) supplemented with 10% (v/v) fetal bovine serum (FBS; Gibco, Vienna, Austria) with 5% CO_2_ at 37 °C.

### RNA preparation and real-time quantitative PCR (RT-qPCR) analysis

RNA was extracted using TRIzol reagent (Invitrogen, CA, USA). For RNase R treatment, total RNA (2 μg) was incubated with or without RNase R (3 U/μg; Epicentre Technologies, Madison, WI, USA) in for 20 min at 37 °C, and the resulting RNA was purified using an RNeasy MinElute cleanup Kit (Qiagen). For cellular RNA fractionation analysis, the cells' nuclear and cytoplasmic fractions were extracted using NE-PER Nuclear and Cytoplasmic Extraction Reagents (Thermo Scientific).

For circBCBM1 and BRD4 expression detection, the cDNAs were synthesized using PrimeScript RT Master Mix (Takara, Dalian, China) according to the manufacturer's instructions. The RT-qPCR was performed using TG Green Premix Ex Taq II kit (Takara, Dalian, China) with a 7500 Real-Time PCR System (Applied Biosystems, Foster City, CA) as previously described 19. *GAPDH* served as an internal reference gene. For miR-125a expression detection, the cDNAs were synthesized using miRNA First Strand cDNA Synthesis kit (Stem-loop Method; Shanghai Sangon Biotech, Shanghai, China) according to the manufacturer's instructions. Then qPCR was performed using MicroRNAs qPCR Kit (SYBR Green Method; Shanghai Sangon Biotech, Shanghai, China). *U6* served as an internal reference gene. All primer sequences are listed in Supplementary Table S1.

### Fluorescence in situ hybridization (FISH)

Cy3-labeled circBCBM1 FISH probe and fluorescein amidite (FAM)-labeled miR-125a probe were designed and synthesized by Guangzhou RiboBio and Invitrogen, respectively. FISH was conducted using Ribo Fluorescent In Situ Hybridization Kit (RiboBio, Guangzhou, China). For cell FISH assay, 231-BR cells were fixed with 4% (w/v) paraformaldehyde (PFA), permeated with 0.5% triton X-100 and hybridized with circBCBM1 probe at 37 °C overnight. 18S and U6 served as reference probes. For tissue FISH assay, the brain slices from brain metastasis mice were fixed, permeated and hybridized with circBCBM1 and miR-125a probes. The hybridization buffer was then gradually eluted with 4×saline-sodium citrate (SSC), 2×SSC and 1×SSC. Nuclei were counterstained with 4,6-diamidino-2-phenylindole (DAPI). The images were acquired on a Leica SP5 confocal microscope (Leica Microsystems, Mannheim, Germany).

### Oligonucleotides, plasmids and transfection

siRNA, miRNA mimics and inhibitors were designed and synthesized by RiboBio (Guangzhou, China) or Sangon Biotech (Shanghai, China). Transfection was performed using Lipofectamine 2000 reagent (Invitrogen, Carlsbad, USA) or riboFECT CP transfection kit (RiboBio, Shanghai, China). The overexpressing and silencing vectors were constructed by HANBIO (Shanghai, China) as described previously 20. All constructs were verified by sequencing. Lentiviral particles carrying the above-mentioned vectors were generated in HEK293T cells. Cells were infected with lentivirus at a multiplicity of infection (MOI) of 30 and screened by puromycin.

### Cell counting kit-8 (CCK8) and colony formation assays

For CCK8 assay, cells were seeded into 96-well plates and cultured overnight, followed by gene silencing or overexpression treatment. After culture, CCK8 (10 μL; Dojindo, Japan) solution was added to each well and incubation for 2 h. Absorbance at 450 nm was measured using a microplate reader (BioTex, Houston, TX, USA). For colony formation assay, cells were seeded into 6-well plates with 1×10^3^ cells/well. After 14 days of culture, cells were fixed with 4% PFA and stained with 0.1% crystal violet.

### Apoptosis detection assay

Apoptosis detection assay was conducted using PE Annexin V Apoptosis Detection Kit I (BD Pharmingen, Franklin Lakes, NJ, USA) according to the manufacturer's procedure. Briefly, cells were harvested, washed and resuspended in 1 × binding buffer, and incubated with 5 μL PE Annexin V and 5 μL 7-AAD for 15 min. The apoptotic cells were assessed using a FACS Calibur flow cytometer (BD Bioscience, San Jose, CA, USA).

### Wound healing and transwell migration assays

For wound healing assay, cells were seeded into 6-well plates and scraped using a pipette tip. Images were obtained using an inverted light microscope at the time points of 0 and 24 h. For transwell migration assay, cells were seeded into the upper chamber. After incubation, the cells were fixed with methanol and stained with crystal violet. Non-migrated cells that remained at the top layer were removed using a cotton swab, and migrated cells at the bottom of the chamber were observed and counted under a light microscope.

### Animal models

Six-week-old female BALB/c nu/nu mice were obtained from Beijing Vital River Laboratory Animal Technology (Beijing, China). The subcutaneous tumor model was generated by subcutaneously injection (s.c) of cells into the right shoulder of the mouse. The breast cancer brain metastasis model was generated by injecting cells into the left ventricle of the mouse heart. Mice were euthanized at the appropriate time points. Mouse brains were collected and stained with hematoxylin and eosin (H&E) as previously described for metastatic nodules count 21. All animal experiments were conducted following the protocols evaluated and approved by the Institutional Animal Care and Use Committee of Liaocheng People's Hospital.

### RNA immunoprecipitation (RIP) assay

RIP assay was conducted using EZ-Magna RIP RNA-Binding Protein Immunoprecipitation Kit (Millipore, Billerica, MA, USA) according to the manufacturer's protocol. Cells were lysed in 100 μl RIP lysis buffer and then diluted with 900 μl RIP immunoprecipitation buffer. The cell suspension was then mixed with magnetic beads conjugated with anti-Argonaute 2 (Ago2) or control anti-IgG antibody and rotated overnight at 4°C. The beads were collected and washed using RIP washing buffer and treated with Proteinase K at 55 °C for 30 min. RNA was extracted using TRIzol reagent (Invitrogen, CA, USA) and analyzed by RT-qPCR.

### Biotinylated RNA pull-down assay

The biotinylated RNA pull-down assay was performed as described previously 20. The 3ʹ-biotinylated miRNA and circRNA probes were designed and synthesized by RiboBio (Guangzhou, China) or GenePharma (Shanghai, China). To pull down circRNA by miRNA, 231-BR cells were transfected with biotinylated miR-125a, miR-1306, miR-34c, miR-26a, miR-10399, miR-661or control miRNA. After 48 h, the cells were washed and lysed. Lysates were incubated with Dynabeads ™ M-280 Streptavidin magnetic beads (Invitrogen, CA, USA) at 4°C for 1.5 h. The bound RNAs were purified using TRIzol for RT-qPCR analysis. To pull down miRNA by circRNA, the biotinylated circBCBM1 probe was incubated with M-280 Streptavidin magnetic beads (Invitrogen, CA, USA) at 4°C for 3 h to generate probe-coated magnetic beads. 231-BR cells were lysed and incubated with probe-coated beads at 4 °C overnight. After washing, the bound RNAs were extracted for RT-qPCR analysis.

### Luciferase reporter assay

HEK293T cells were seeded in 96-well plates and cultured for 24 h. The cells were co-transfected with a mixture of miRNA mimics and luciferase reporter vectors containing BRD4 3′-UTR sequences. After 48 h, the luciferase activity was determined using a dual-luciferase reporter assay system (Promega, Madison, WI, USA) following the manufacturer's protocol. Renilla luciferase activity was normalized to firefly luciferase activity and presented as the percentage of the control.

### Western blotting analysis

Proteins were extracted in RIPA lysis buffer (P0013B, Beyotime) and the concentration was determined using a BCA Protein assay kit (P0010S, Beyotime). Proteins were separated on sodium dodecyl sulfate-polyacrylamide gels (SDS-PAGE) and then transferred to polyvinylidene fluoride (PVDF) membranes. The membranes were blocked with 5% non-fat dry milk and then incubated overnight with primary antibodies, including anti-BRD4 (ab128874, Abcam), anti-MMP9 (ab38898, Abcam), anti-Shh (2207s, CST), anti-Gli1 (2643s, CST) and anti-GAPDH (sc-32233, Santa Cruz Biotechnology). Membranes were then incubated with horseradish peroxidase (HRP)-conjugated goat anti-mouse/rabbit IgG secondary antibody at room temperature. The blots were detected by chemiluminescence and imaged on an AlphaView analysis system (ProteinSimle, USA). The quantification of individual protein bands was assessed by densitometry using ImageJ software.

### mRNA sequencing

Total RNA from 231-BR cells or MDA-MB-231 cells was extracted using TRIzol reagent (Invitrogen, CA, USA). Ribosomal RNA was removed by Epicentre Ribo-zerorRNA Removal Kit (Epicentre, USA). The sequencing libraries were generated by NEBNext Ultra Directional RNA Library Prep Kit (NEB, Beverly, USA) following the manufacturer's protocol. Briefly, RNA was fragmented and first-strand cDNA was synthesized using random hexamer primers and M-MuLV ReverseTranscriptase (RNaseH-). The second strand cDNA synthesis was performed using DNA Polymerase I and RNase H, with dUTP replacing dTTP in the reaction buffer. After adenylation at the 3' ends of cDNA fragments, NEBNext Adaptor with a hairpin loop structure was ligated for hybridization. The library fragments were purified with the AMPure XP system (Beckman Coulter, Beverly, US) and then treated by 3 μL USER Enzyme (NEB, Beverly, USA) at 37 °C for 15 min followed by 95 °C for 5 min. After PCR amplification, the products were purified and library quality was assessed on Agilent Bioanalyzer 2100 (Agilent Technologies, CA, USA). The index-coded library was clustered on cBot Cluster Generation System (Illumina Inc., San Diego, CA), and was sequenced on Illumina HiSeq 2500 platform and 125 bp paired-end reads were generated. An index of the reference genome was built and paired-end clean reads were mapped to the reference genome using HISAT2. The mapped reads of each sample were assembled by StringTie. Differential expression of replicated count data was examined using the edgeR software package.

### Immunohistochemistry (IHC) staining

IHC staining was performed as described previously^19^. Briefly, formalin-fixed tissue specimens were cut as serial 5-µm sections. The sections were deparaffinized, hydrated and boiled in citrate buffer (pH 6.0) to retrieve epitopes. After quenching endogenous peroxidase with 3% hydrogen peroxide and blocking non-specific binding with 10% horse serum, the sections were incubated with primary antibodies, including anti-BRD4 (ab128874, Abcam) and anti-MMP9 (ab38898, Abcam). Subsequently, the sections were rinsed and incubated with appropriate biotinylated secondary antibodies and incubated with peroxidase-conjugated streptavidin. Finally, the sections were reacted with 3,3'-Diaminobenzidine tetrahydrochloride (DAB) solution for 5 min, followed by counterstaining with hematoxylin. The sections were viewed with a light microscope.

### Statistical analysis

All *in vitro* experiments were repeated at least three times. The quantitative data were presented as means ± standard error of the mean (SEM) and analyzed by *t*-test or one-way ANOVA. Pearson correlation analysis was used to analyze the relative expression of circBCBM1 and miR-125a. BMFS was defined as the length of time from the date of surgery to brain metastasis. BMFS was calculated with Kaplan-Meier estimates and analyzed with the log-rank test. All statistical analyses were performed using SPSS18 software (SPSS Inc., Chicago, USA).* P* < 0.05 was considered statistically significant.

## Results

### Characterization of circBCBM1 in 231-BR cells

**Figure 1:**
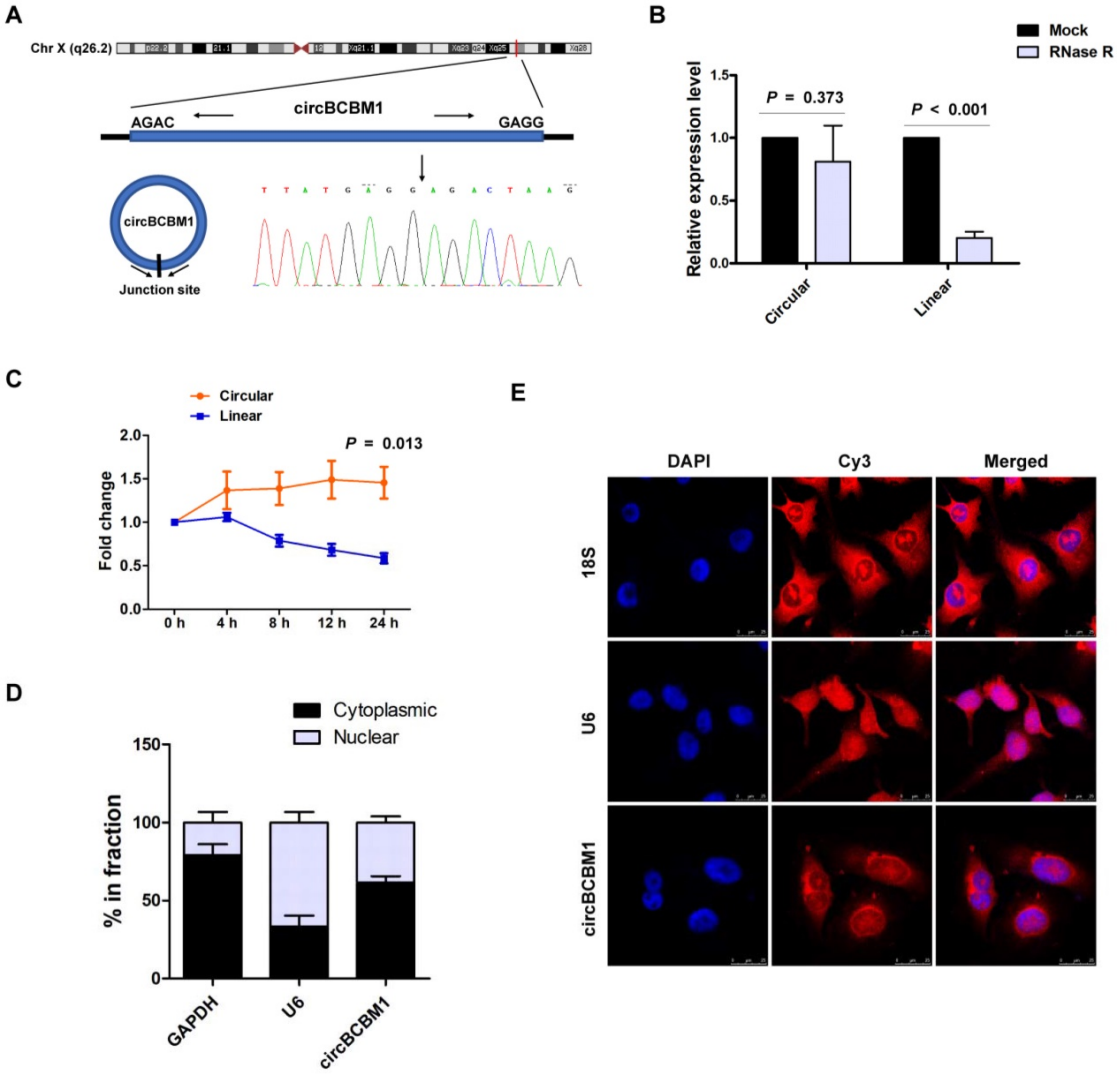
Characterization of circBCBM1 in 231-BR cells. (A) The schematic diagram of genomic location and splicing pattern of circBCBM1. The splice junction was verified by Sanger sequencing. (B) RT-qPCR analysis for the abundance of circBCBM1 and its linear counterpart after treatment with RNase R in 231-BR cells. The amount of circBCBM1 and its linear counterpart were normalized to the value measured in the mock treatment. (C) The expression level of circBCBM1 and its linear counterpart after treatment with Actinomycin D (2.5 μg/ml) at the indicated time points in 231-BR cells. (D) Cellular RNA fractionation analysis. CircBCBM1 was mainly located in the cytoplasm of 231-BR cells. (E) RNA fluorescence in situ hybridization (FISH) for circBCBM1. Nuclei were stained with 4,6-diamidino-2-phenylindole (DAPI). Scale bar, 25 μm. Data are presented as means ± SEM (B-D).

We previously identified the circRNA expression profile of brain metastatic breast cancer cell line 231-BR, in comparison with its parental nonspecific metastatic cell line MDA-MB-231, using RNA-seq 17. CircBCBM1 was one of the most significantly upregulated circRNAs in 231-BR cells. CircBCBM1 is derived from a long non-coding RNA region within the FIRRE locus (chrX: 130883333 - 130928494), which is located on chromosome Xq26.2. The exact size of spliced mature circBCBM1 is 1096 bp. The back-spliced junction of circBCBM1 was amplified with outward-facing primers and validated by Sanger sequencing (Figure 1A). Its resistance to RNase R exonuclease digestion further confirmed that it exists in a circular form (Figure 1B).

We next investigated the stability and localization of circBCBM1. After treatment with transcription inhibitor Actinomycin D, the expression level of the circular RNA isoform of circBCBM1 remained stable, while its linear counterpart level was remarkably decreased (Figure 1C). Cellular RNA fractionation analysis showed that circBCBM1 was predominately localized within cytoplasm instead of nuclei in 231-BR cells (Figure 1D), which was further confirmed by FISH (Figure 1E). Taken together, these results implied that circBCBM1 is a stable and cytoplasmic circRNA.

### CircBCBM1 promotes proliferation and migration of 231-BR cells *in vitro*

**Figure 2:**
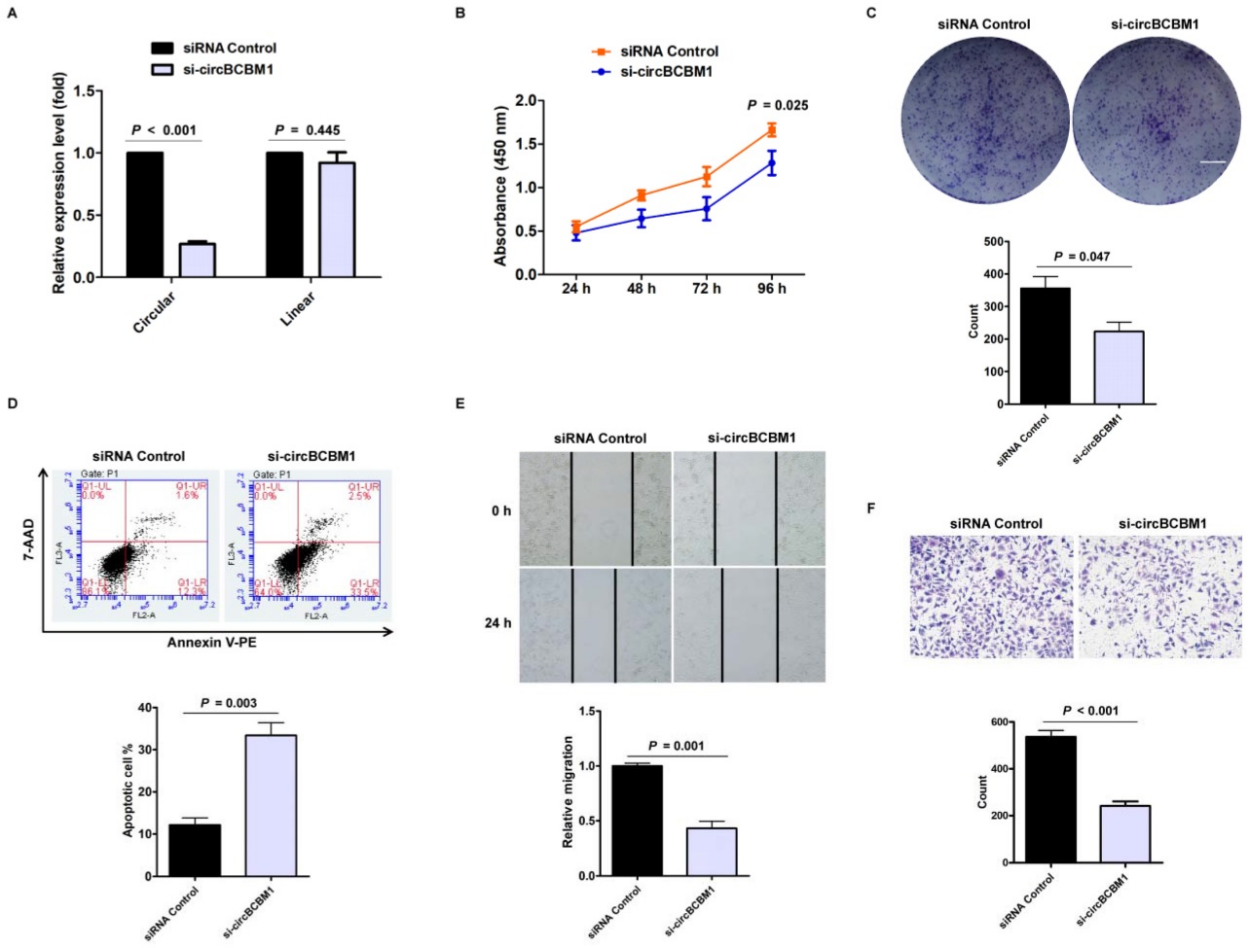
Silencing of circBCBM1 inhibits proliferation and migration and induces apoptosis of 231-BR cells* in vitro*. 231-BR cells were either transfected with si-circBCBM1 or siRNA Control. (A) RT-qPCR for the expression analysis of circBCBM1 and its linear counterpart. The relative expression levels were normalized to the values in the siRNA-control group. (B) CCK-8 assay. (C) Colony formation assay. Scale bar, 5 mm. (D) Annexin V-PE/ 7-AAD staining and FACS quantified the apoptotic cells percentage. (E) Wound-healing assay. (F) Transwell migration assay. Data are presented as means ± SEM (A-F). si-circBCBM1, siRNA against circBCBM1; siRNA Control, siRNA for negative control.

To evaluate the biological functions of circBCBM1, we designed specific siRNA oligonucleotides to target the unique backsplice junction. A nonspecific siRNA sequence served as control. As expected, the backsplice junction-specific siRNA (si-circBCBM1) reduced circBCBM1 level but did not affect the linear counterpart level (Figure 2A). CCK8 assay showed that circBCBM1 silencing significantly suppressed cell proliferation (*P* = 0.025; Figure 2B). Colony formation assay showed that circBCBM1 silencing significantly inhibited colony-forming of 231-BR cells (*P* = 0.047; Figure 2C). Annexin V-PE/ 7-AAD staining demonstrated that circBCBM1 silencing significantly promoted cellular apoptosis (*P* = 0.003; Figure 2D). Wound healing and transwell migration experiments revealed that circBCBM1 silencing significantly inhibited the migration of 231-BR cells (*P* < 0.05; Figure 2E and 2F). Moreover, the inhibitory effect of circBCBM1 silencing on cell proliferation, clone formation and migration capabilities were further confirmed by another independent siRNA targeting the back-splicing region (si-circBCBM1#2) (Supplementary Figure S1A-1D).

To further verify the role of circBCBM1, circBCBM1-overexpressing 231-BR cells were obtained by transfecting with circBCBM1-expressing plasmids. As shown in Supplementary Figure S2A, the relative expression level of the circular RNA isoform of circBCBM1 was dramatically increased (*P* = 0.032), while its linear counterpart did not increase significantly (*P* = 0.388). CircBCBM1 overexpression prominently increased the proliferation, clone formation and migration capabilities of 231-BR cells (Supplementary Figure S2B-S2E). Furthermore, the enhanced effects of circBCBM1 overexpression on the proliferation and migration capabilities were further confirmed in MDA-MB-231 cells (Supplementary Figure S3A-3C). Collectively, these findings indicate that circBCBM1 promotes cell proliferation and migration of 231-BR cells* in vitro*.

### CircBCBM1 facilitates tumor growth and brain metastasis *in vivo*

**Figure 3:**
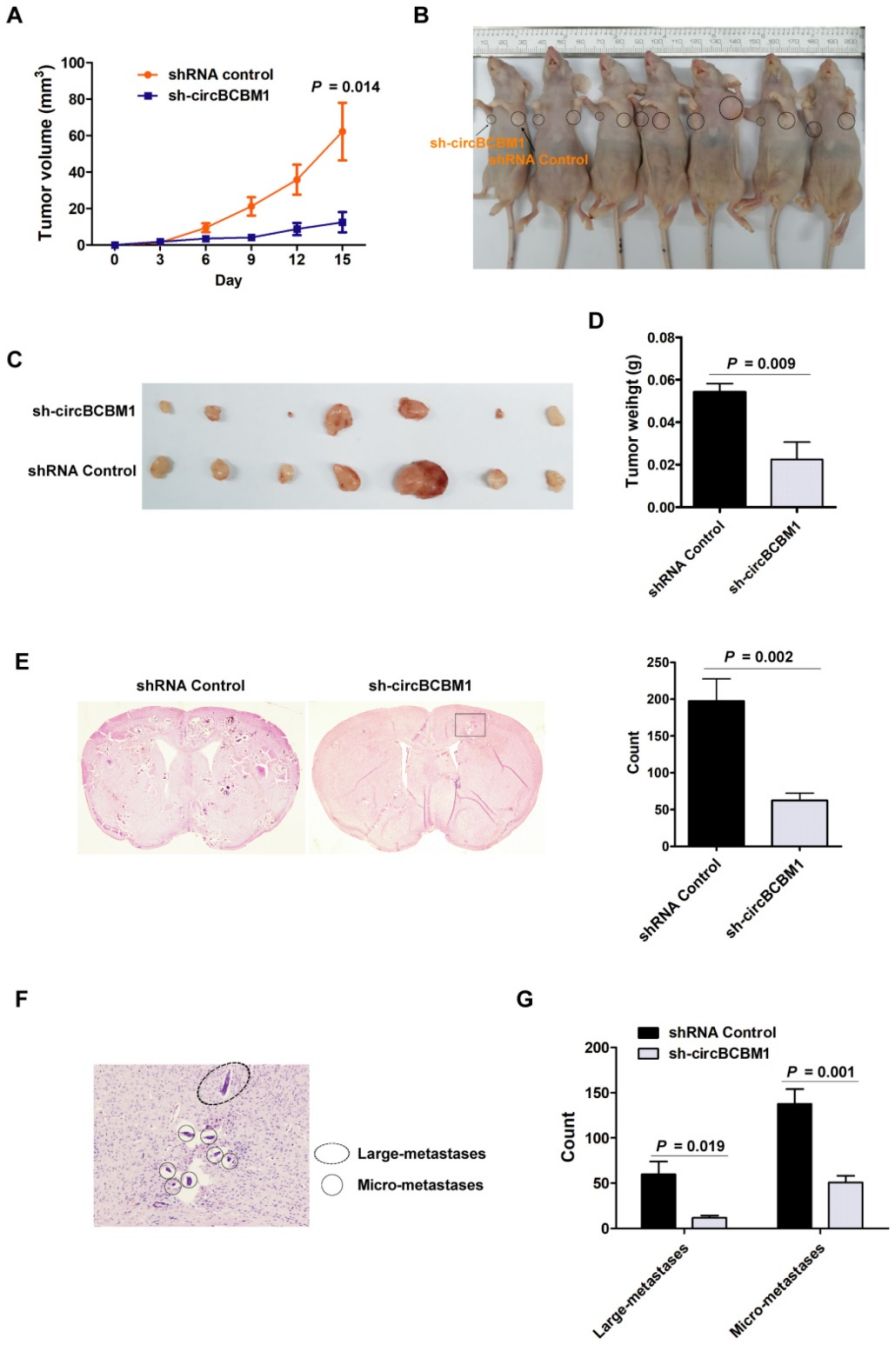
Silencing of circBCBM1 inhibits growth and brain metastasis of 231-BR cells* in vivo*. Nude mice were subcutaneously injected 231-BR cells with stable transfection of negative control (shRNA Control) or shRNA-circBCBM1 (n = 7) (A-D). (A) Tumor growth curves of subcutaneous models. (B) Images of the subcutaneous xenograft models on the 15th day. (C) Images of the dissected subcutaneous tumors. (D) Weights of the dissected subcutaneous tumors. Brain metastasis model was generated by injection of 231-BR cells (2 × 10^5^ per mouse) with stable transfection of negative control (shRNA Control) or shRNA-circBCBM1 into the left cardiac ventricle of mouse heart. After 4 weeks, the brains were collected and metastatic nodules were counted after H&E staining (n = 6) (E-G). (E) Representative H&E images and quantification of brain metastases. (F) An enlargement of the corresponding area (black rectangle) of H&E image in (E). The metastasis nodules were categorized into large- (> 50 μm^2^) and micro-metastases (≤ 50 μm^2^). Ovals, large-metastases; Circles, micro-metastases. (G) Quantification of the counts of large-metastases and micro-metastases. Data are presented as means ± SEM (A, D, E and G).

Based on our *in vitro* findings that circBCBM1 was involved in cell proliferation and migration, we further explored its role in tumor growth and brain metastasis *in vivo*. Tumor growth was researched by subcutaneously injecting 231-BR cells with circBCBM1 stable knockdown (sh-circBCBM1) into nude mice. The results revealed that circBCBM1 knockdown significantly decreased tumor volumes and weights *in vivo* (*P* < 0.05; Figure 3A-3D).

Brain metastasis was explored by injecting 231-BR cells with circBCBM1 stable knockdown (sh-circBCBM1) into the left cardiac ventricle of nude mice. Four weeks after injection, the mouse brains were excised for hematoxylin and eosin (H&E) staining. The total count of brain metastasis nodules was found significantly decreased in the sh-circBCBM1 group compared with those in the control group (*P* = 0.002; Figure 3E). We further categorized the metastasis nodules into large- (> 50 μm^2^) and micro-metastases (≤ 50 μm^2^) (Figure 3F). Compared with the control group, the counts of the large- and micro-metastases in the sh-circBCBM1 group were both significantly decreased (*P* = 0.019 and 0.001, respectively; Figure 3G).

Moreover, we assessed the effect of circBCBM1 stable overexpressing on brain metastasis of 231-BR cells. As shown in Supplementary Figure S4A and S4B, both large- and micro-metastases in the circBCBM1-overexpressing group were increased relative to those in the control group (*P* = 0.026 and 0.006, respectively). We also confirmed the biological roles of circBCBM1 using circBCBM1 stable overexpressing MDA-MB-231 cells *in vivo*. The results indicated that circBCBM1 overexpression significantly increased tumor volumes and weights (*P* < 0.05; Supplementary Figure S5A-S5D). Compared with the control group, the proportion of mice with brain metastases in the circBCBM1 overexpression group was significantly increased (*P* = 0.041; Supplementary Figure S5E and S5F). Collectively, these findings demonstrate that circBCBM1 facilitates tumor growth and brain metastasis *in vivo.*

### CircBCBM1 serves as a miR-125a sponge in 231-BR cells

**Figure 4:**
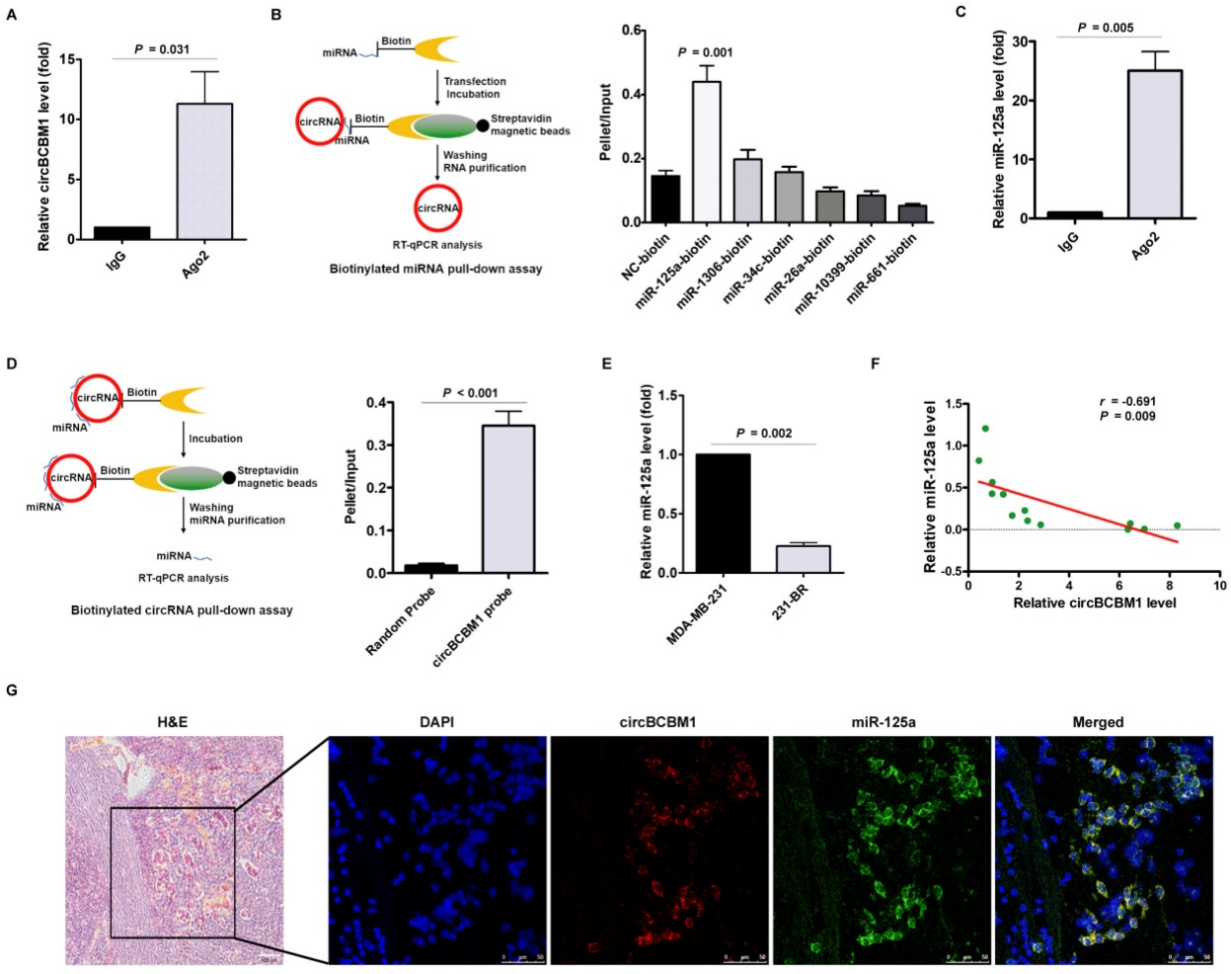
CircBCBM1 directly interacts with miR-125a in 231-BR cells. (A) RNA immunoprecipitation (RIP) and RT-qPCR assays were conducted to analyze the binding of circBCBM1 to Ago2 protein. (B) Biotinylated miRNA pull-down assay. RNA was affinity-isolated by biotinylated miR-125a, miR-1306, miR-34c, miR-26a, miR-10399, miR-661 or the negative control miRNA, and the circBCBM1 and GAPDH mRNA levels were quantified by RT-qPCR. The relative level of circBCBM1 was normalized to input. (C) RIP and RT-qPCR assays were performed to analyze the binding of miR-125a to Ago2 protein. (D) Biotinylated circBCBM1 pull-down assay. RNA was affinity-isolated by biotinylated circBCBM1 or the control probe, and the circBCBM1 and U6 levels were analyzed by RT-qPCR. The relative level of miR-125a was normalized to input. (E) The relative level of miR-125a in 231-BR cells versus the parental MDA-MB-231 cells. (F) The expression correlation analysis between circBCBM1 and miR-125a in 13 pairs of breast cancer and adjacent normal breast tissues. (G) FISH assay of circBCBM1 and miR-125a in the brain slices of brain metastasis mice. Left panel, H&E staining. Blue, DAPI; Red, circBCBM1; Green, miR-125a. Scale bar: H&E, 100 μm; FISH, 50 μm. Data are presented as means ± SEM (A-E).

Having determined the essential role of circBCBM1 in breast cancer brain metastasis, we tried to get insight into the mechanisms of circBCBM1 regulation. Given that circRNAs can act as competing endogenous RNA sponges to interact with miRNAs and influence their activity, we explored whether circBCBM1 functions as a miRNA sponge in 231-BR cells by conducting an RIP assay with Ago2 antibody. As shown in Figure 4A, endogenous circBCBM1 was significantly enriched by anti-Ago2 compared with the control IgG antibody (*P* = 0.031), suggesting that circBCBM1 is able to bind to Ago2 and miRNAs.

To dissect which miRNA circBCBM1 binds to, we conducted bioinformatics analysis using miRanda and identified 6 candidate miRNAs, including miR-125a, miR-1306, miR-34c, miR-26a, miR-10399 and miR-661 (Supplementary Table S2). In miRNA pull-down assay using biotin-coupled miRNA mimics, circBCBM1 was only efficiently enriched by miR-125a, but not by the other five miRNAs (Figure 4B). In the RIP assay, miR-125a was efficiently pulled down by anti-Ago2 antibody (Figure 4C). An inverse affinity isolation assay using a biotin-labeled circBCBM1 probe also confirmed that circBCBM1 probe, but not the random probe, bound to miR-125a (Figure 4D). Compared with the parental MDA-MB-231 cells, the relative expression level of miR-125a was significantly lower in 231-BR cells (*P* = 0.002; Figure 4E). The expression correlation between circBCBM1 and miR-125a was analyzed by RT-qPCR. The results revealed that miR-125a levels were inversely correlated with circBCBM1 levels in breast cancer tissues (*r* = -0.691, *P* = 0.009; Figure 4F). Moreover, a double FISH assay indicated circBCBM1 and miR-125a were co-localized in the brain slices of brain metastasis mice (Figure 4G). Collectively, these findings provide evidence that circBCBM1 acts as a sponge for miR-125a in 231-BR cells.

### BRD4 is the downstream target of miR-125a

**Figure 5:**
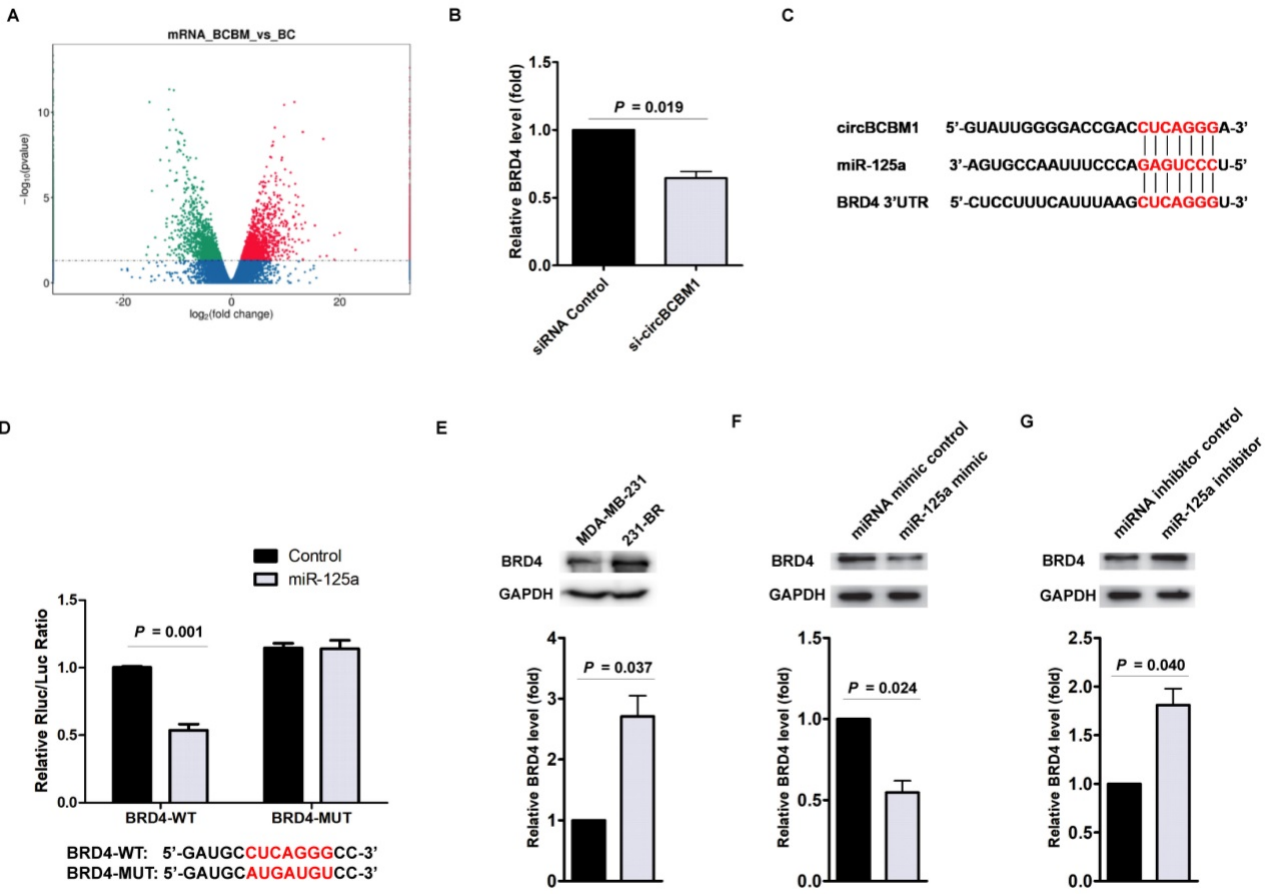
BRD4 is the downstream target of the circBCBM1/miR-125a axis. (A) Volcano plots assessing the differentially expressed mRNAs between 231-BR (BCBM) and its parental MDA-MB-231 cell (BC) groups. (B) RT-qPCR assays analyzed the expression of BRD4 in 231-BR cells after treatment with si-circBCBM1. (C) Putative miR-125a binding site in BRD4. The potential complementary residues are shown in red. (D) Relative luciferase activity of BRD4 wild-type (BRD4-WT) and 3ʹ-UTR mutant (BRD4-MUT) constructs co-transfected with miR-125a mimics or miRNA negative control. (E) Western blot analysis of BRD4 expression in 231-BR cells versus the parental MDA-MB-231 cells. (F and G) Western blot assays examined the expression levels of BRD4 in 231-BR cells transduced with miR-125a mimics (F) or miR-125a inhibitors (G). MiRNA mimic control (F) and miRNA inhibitor control (G) were used as the miRNA mimic and inhibitor negative control, respectively. Data are presented as means ± SEM (B, D-G).

To elucidate the molecular mechanisms by which circBCBM1/miR-125a is involved in breast cancer brain metastasis, we predicted potential target genes of miR-125a using TargetScan. On the other hand, RNA-seq was performed to identify differentially expressed mRNAs between 231-BR cells (BCBM group) and MDA-MB-231 cells (BC group) (Figure 5A). Based on the integrated analysis of bioinformatics and differentially expressed mRNAs, we selected candidate potential target genes of miR-125a, including *BRD4*, *HDDC3* and *TXNRD1*. We next examined the effect of circBCBM1 on the candidate target genes' expression. RT-qPCR results showed only *BRD4* was significantly downregulated after circBCBM1 silencing in 231-BR cells (Figure 5B and Supplementary Figure S6A and S6B). Thus, we selected *BRD4* for further study.

The predicted binding site between miR-125a and *BRD4* was shown in Figure 5C. We next cloned the wild-type and mutant (with a mutated predicted miR-125a binding site) 3′-UTR of *BRD4* mRNA and performed dual-luciferase reporter assay. As shown in Figure 5D, 231-BR cells co-transfected with miR-125a mimic and pmiR-GLO plasmid with wild-type *BRD4* 3ʹ-UTR showed decreased luciferase activity (*P* = 0.001), whereas this effect was not observed in the mutated *BRD4* 3ʹ-UTR. This result suggested that the predicted binding element was essential for miR-125a binding to the 3′-UTR of *BRD4*.

Compared with MDA-MB-231 cells, the relative expression level of BRD4 was significantly higher in 231-BR cells (*P* = 0.037; Figure 5E). Moreover, in 231-BR cells, miR-125a mimics reduced BRD4 expression (*P* = 0.024; Figure 5F), whereas miR-125a inhibitors improved BRD4 expression at the protein level (*P* = 0.040; Figure 5G). Taken together, these findings reveal that miR-125a bind to 3′-UTR of BRD4 and directly downregulate its expression.

### CircBCBM1 promotes cell migration via circBCBM1/miR-125a/BRD4 axis in 231-BR cells

**Figure 6:**
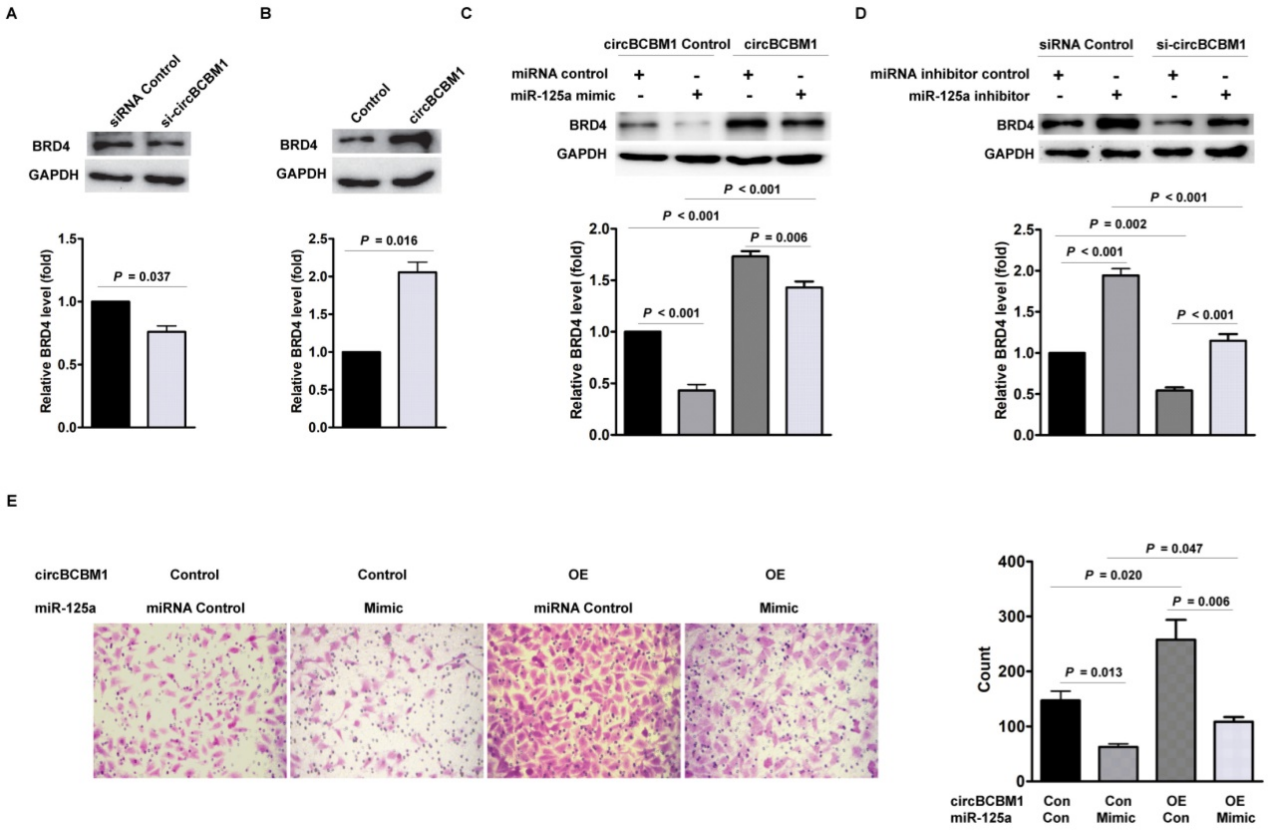
CircBCBM1 promotes cell migration via circBCBM1/miR-125a/BRD4 axis in 231-BR cells. (A) Western blot analysis of BRD4 expression in 231-BR cells after treatment with si-circBCBM1. (B) Western blot analysis of BRD4 expression in 231-BR cells transfected with control vector (Control group) or circBCBM1 over-expression plasmid (circBCBM1 group). (C) Transduction of 231-BR cells with circBCBM1 overexpressing lentivirus significantly increased miR-125a-inhibited BRD4 expression as determined by western blot analysis. (D) Transduction of 231-BR cells with circBCBM1 siRNA significantly inhibited miR-125a inhibitor induced BRD4 expression as determined by western blot analysis. (E) CircBCBM1 overexpression rescued the migration of 231-BR cells after transfection with miR-125a mimics as determined by transwell migration assay. OE: overexpression; Con: control. Data are presented as means ± SEM (A-E).

Having determined that BRD4 is the downstream target of miR-125a, we hypothesized that circBCBM1 promoted cell migration via circBCBM1/miR-125a/BRD4 axis. As shown in Figure 6A, circBCBM1 silencing decreased BRD4 expression at the protein level (*P* = 0.037). On the contrary, circBCBM1 overexpression increased the protein level of BRD4 (*P* = 0.016; Figure 6B). To further verify that miR-125a acts as a mediator of circBCBM1 to control the expression of BRD4, we conducted rescue experiments. In 231-BR cells, circBCBM1 overexpression significantly lessened the decreased expression of BRD4 induced by miR-125a mimics (*P* < 0.001; Figure 6C). On the other hand, circBCBM1 knockdown attenuated the inductive effects of miR-125a inhibitors on BRD4 expression (*P* < 0.001; Figure 6D). More importantly, transwell migration experiments showed that miR-125a mimics abolished the enforced cell migration induced by circBCBM1 (Figure 6E). Collectively, these findings suggest that circBCBM1 promotes 231-BR cells' migration via circBCBM1/miR-125a/BRD4 axis.

### BRD4 promotes MMP9 expression through the SHH signaling pathway in 231-BR cells

**Figure 7:**
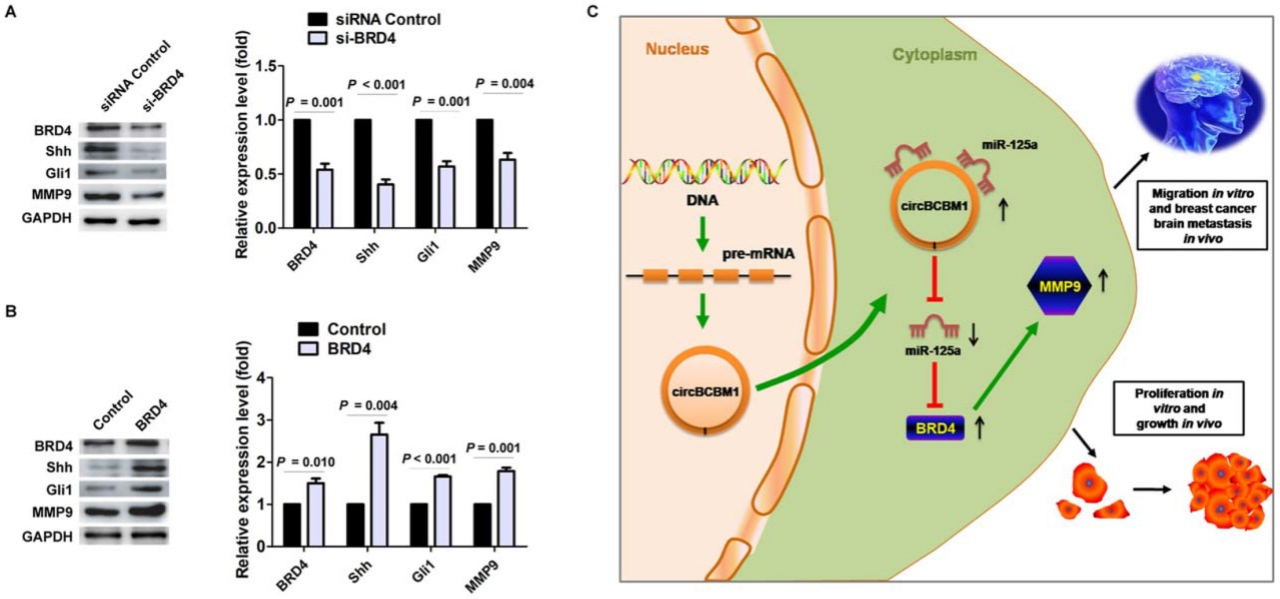
BRD4 promotes MMP9 expression through SHH signaling pathway in 231-BR cells. (A and B) Western blot analyses of BRD4, Shh, Gli and MMP9 expression in 231-BR cells transfected with si-BRD4 (A) or BRD4 over-expression plasmid (BRD4 group) (B). The relative expression level was normalized to that of the siRNA control group (A) or control vector group (B). Data are presented as means ± SEM. (C) Schematic representation of the mechanism and function of circBCBM1 in breast cancer brain metastasis.

Studies have previously demonstrated that MMP9 contributed to breast cancer brain metastasis by promoting cells' trans-endothelial migration and permeability across the blood-brain barrier (BBB), and its expression level was correlated with breast cancer brain metastasis-free survival 22-25. Our findings confirmed the expression of MMP9 in the human breast cancer brain metastasis samples by IHC staining (Supplementary Figure S7). Moreover, BRD4 was also observed obvious expression in the samples and roughly overlapped in the spatial distribution with MMP9 (Supplementary Figure S7). We next sought to examine the effect of BRD4 expression on MMP9 level in 231-BR cells. As shown in Figure 7A and 7B, BRD4 silencing resulted in decreased MMP9 expression (*P* = 0.004), while BRD4 overexpression led to increased MMP9 expression (*P* = 0.001). Mechanistically, a previous study demonstrated that BRD4 regulated MMP-9 expression through the SHH signaling pathway in hepatocellular carcinoma cells 26. In the context of breast cancer brain metastasis, we also found BRD4 silencing caused downregulation of two downstream target molecules, Shh and Gli, while BRD4 overexpression improved their expression (Figure 7A and 7B), suggesting that BRD4 may promote MMP9 expression through SHH signaling pathway in 231-BR cells. Overall, these findings demonstrated that circBCBM1 sponged miR-125a and upregulated BRD4, resulting in the upregulation of MMP9, and thereby promoting 231-BR cell proliferation and migration *in vitro*, and growth and brain metastasis *in vivo* (Figure 7C).

### CircBCBM1 may act as a potential diagnostic and prognostic biomarker for breast cancer brain metastasis

**Figure 8:**
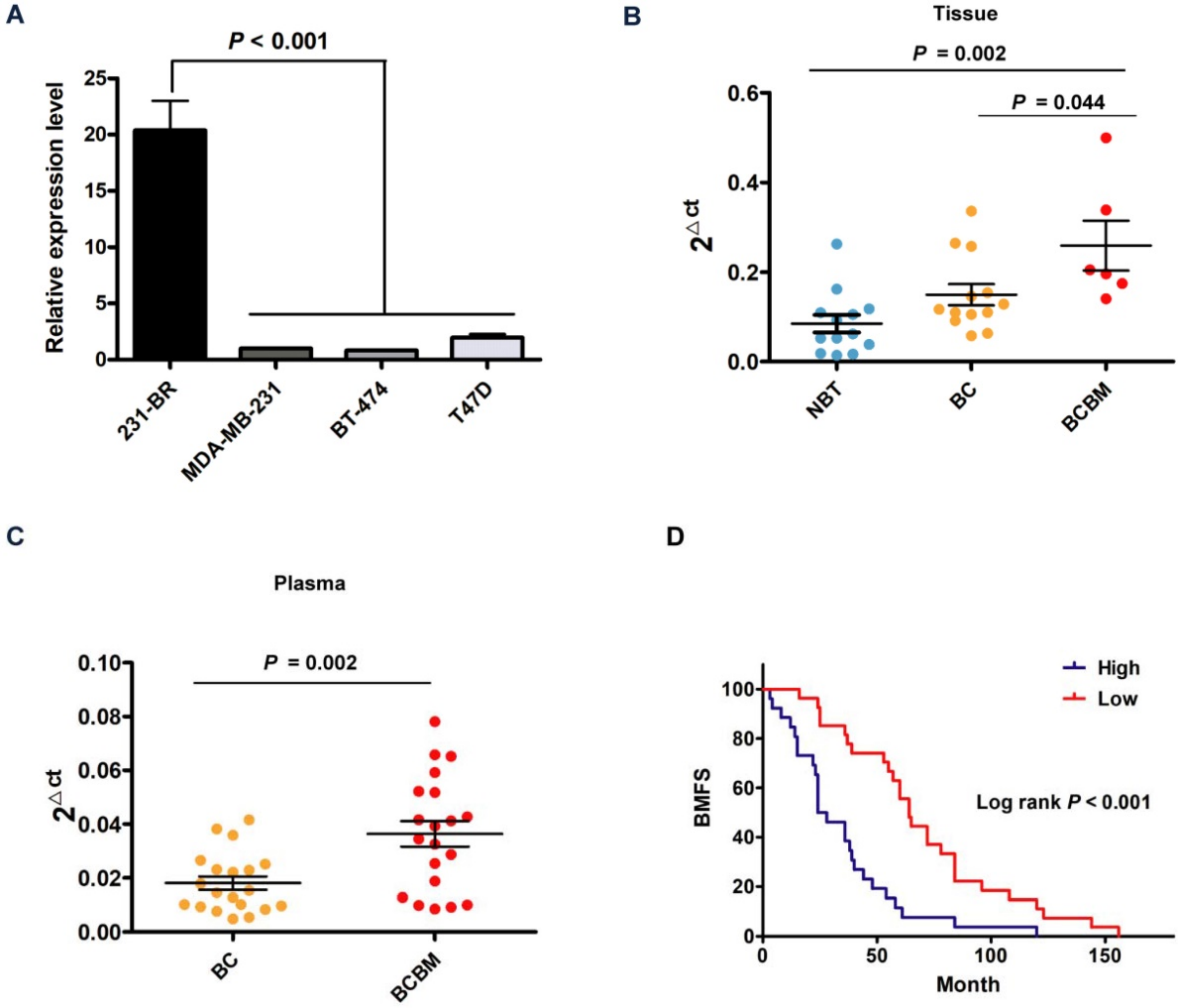
The potential diagnostic and prognostic value of circBCBM1. (A) RT-qPCR analysis of circBCBM1 expression in 231-BR cells versus other breast cancer cells (MDA-MB-231, BT-474 and T47D). The relative expression level was normalized to that of MDA-MB-231 cells. (B and C) RT-qPCR analyses of circBCBM1 expression level in tissue (B) and plasma (C) samples. For (B), NBT, adjacent normal breast tissues, n = 13; BC, breast cancertissues, n = 13; BCBM, breast cancer brain metastasis tissues, n = 6. For (C), BC, breast cancer plasmas, n = 20; BCBM, breast cancer brain metastasisplasmas, n = 20. Data are presented as means ± SEM (A-C). (D) Kaplan-Meier analysis for brain metastasis-free survival (BMFS) of 53 BCBM patients. Patients were divided into two groups based on the expression of circBCBM1 in the patients' primary tumors. *P-*value was calculated using the log-rank test.

Considering the vital role and underlying mechanism of circBCBM1 in breast cancer brain metastasis, it might be used as a diagnostic and prognostic biomarker. We examined the expression levels of circBCBM1 in various breast cancer cell lines and found circBCBM1 was significantly upregulated in brain metastatic 231-BR cells compared with the other cell lines (MDA-MB-231, BT-474 and T47D) (*P* < 0.001; Figure 8A). We also examined the expression level of circBCBM1 in tissue samples, which showed that circBCBM1 was markedly upregulated in breast cancer brain metastasis tissues compared with other breast cancer and normal breast tissues (*P* = 0.044 and 0.002, respectively; Figure 8B). Given that blood is the most common sample and liquid biopsy is a less invasive laboratory technique 27, we attempted to figure out the circBCBM1 level in plasma of breast cancer patients with or without brain metastases. By RT-qPCR, we found circBCBM1 was detectable in plasma samples. In agreement with the results from tissue samples, circBCBM1 level was significantly higher in brain metastasis patients' plasma than those in patients without metastases (*P* = 0.002; Figure 8C), indicating its translational potential as a biomarker for breast cancer brain metastasis patient identification.

To evaluate whether circBCBM1 could serve as a prognostic marker, the BMFS curve was plotted by Kaplan-Meier estimates according to the circBCBM1 expression level in their primary site counterparts. As shown in Figure 8D, breast cancer patients with a high expression level of circBCBM1 had significantly shorter BMFS (24 months *vs* 64 months, *P* < 0.001), suggesting that the circBCBM1 level in primary tumor tissue may act as a potential biomarker for predicting the risk of brain metastasis in breast cancer patients.

## Discussion

Brain metastasis is a fatal neurological complication accompanying systemic cancers. Identification of key molecules and mechanisms in breast cancer brain metastasis is a prerequisite for the development of diagnostic and prognostic biomarkers and therapeutic targets for future innovative treatments. In this study, we elucidated a novel mechanism that breast cancer brain metastasis may be regulated by circBCBM1/miR-125a/BRD4 signaling axis. We found upregulation of circBCBM1 stimulated the proliferation and migration of 231-BR cells* in vitro* and growth and brain metastasis *in vivo*. Moreover, circBCBM1 was upregulated in brain metastatic breast cancer cells, clinical patients' tissue and plasma samples, and a high circBCBM1 level was associated with short BMFS. To the best of our knowledge, this is the first report that thoroughly investigated the expression, function and molecular mechanism of circBCBM1 in human breast cancer brain metastasis.

CircRNAs belongs to a novel class of ncRNAs that have attracted numerous research attention. Mounting evidence demonstrates that circRNAs play an important role in tumorigenesis and progression 28. Our findings revealed that circBCBM1 was upregulated in the breast cancer brain metastasis tissues, and a high expression level of circBCBM1 in primary cancerous tissues was correlated with short BMFS, suggesting that circBCBM1 may serve as a potential biomarker for predicting the risk of brain metastasis in breast cancer patients. CircRNAs are more stable than their linear counterparts due to their covalent closed cyclic structure and therefore are enriched in plasma, cell-free saliva, and even in circulating exosomes, which may predict the occurrence of cancer and other diseases 7, 29. In the present study, we found circBCBM1 was detectable in plasma samples, and its level was significantly increased in breast cancer brain metastasis patients' plasma than those in patients without metastases, suggesting that circBCBM1 may serve as a potential circulating biomarker for distinguishing breast cancer patients with brain metastases from those without metastases.

Apart from acting as diagnostic or prognostic biomarkers, circRNAs may also be developed into therapeutic targets 30, 31. Huang et al. reported that specific blockage of circHIPK2 may inhibit astrocyte activation in the context of drug abuse and neuroinflammatory disorders 32. Yang et al. found that circPTK2 exerted a critical role in the growth and metastasis of colorectal cancer (CRC) and may serve as a therapeutic target for CRC metastasis 16. Our findings showed circBCBM1 facilitated cell proliferation and migration *in vitro* and growth and brain metastasis *in vivo*. These results suggested that circBCBM1may act as an oncogene, and could be envisioned as a novel therapeutic target for breast cancer brain metastasis.

Based on accumulating evidence, the most explored function of circRNAs is regulating gene expression through ´sponging´ other gene expression regulators, in particular miRNA 33. For example, circPTCH1 could act as a sponge for miR-485-5p to promote invasion and metastasis in renal cell carcinoma 34. Hsa_circ_0000326 interacted with miR-338-3p to facilitate lung adenocarcinoma progression 35. Herein, we predicted and verified that circBCBM1 contained a direct binding site of miR-125a, which suggested that circBCBM1 might serve as a miR-125a sponge to promote proliferation and migration of 231-BR cells. Recent studies have shown that circRNAs also functions as scaffolding protein or translational templates 36. Further investigation is still required to elucidate whether circBCBM1 is involved in these biological processes.

MiRNAs are a class of non-coding small RNAs that post-transcriptionally regulate gene expression by binding to specific mRNA targets and promoting mRNA degradation and/or inhibiting mRNA translation 37. Recently, miR-125a has been reported as a tumor suppressor that was significantly downregulated in multiple cancer types, such as non-small cell lung cancer 38, gastric cancer 39, bladder cancer 40, etc. In our study, BRD4 was predicted as the candidate target gene of miR-125a by Targetscan and was verified by dual-luciferase reporter assay. The rescue experiments further revealed that circBCBM1 promotes 231-BR cells' migration via circBCBM1/miR-125a/BRD4 axis.

BRD4, a member of the bromodomain and extra-terminal (BET) family, is a crucial epigenetic regulator. Multiple studies reported that BRD4 elevated oncogenic protein levels and accelerated carcinogenesis and progression 41, 42. Wang et al. reported that BRD4 regulated MMP-9 expression through SHH signaling pathway in hepatocellular carcinoma 26. Consistent with this finding, our data showed BRD4 regulated MMP-9 expression and key signaling molecules of SHH pathway. Mounting evidence indicates BBB serves as a strong barrier against cancer cell invasion to the brain. The permeability of BBB is known to be increased by MMP9 overexpression and secretion 25. Therefore, we speculate that BRD4 may be involved in breast cancer brain metastasis by upregulating MMP9, resulting in BBB disruption, and thereby promoting the initiation and progression of brain metastasis. However, the exact mechanism requires further illustration.

In conclusion, our study revealed that circBCBM1 promoted 231-BR cell proliferation and migration *in vitro* and growth and brain metastasis *in vivo*. Mechanistically, circBCBM1 increased BRD4 expression via acting as a miR-125a sponge. Importantly, circBCBM1 was upregulated in brain metastasis patients' tissue and plasma samples, and a high expression level of circBCBM1 in primary cancerous tissues was associated with short BMFS. Taken together, our study clarified that circBCBM1 accelerated breast cancer brain metastasis via circBCBM1/miR-125a/BRD4 axis, and provided innovative candidate targets for breast cancer brain metastasis diagnosis and therapy.

## Supplementary Material

Supplementary figures and tables.

## Authors' contributions

B.F., W.L., C.Z., P.L., L.W., L.P., K.L., P.C., M.M., Y.W., A.Z., W.T. and M.A. performed the experiments and analyses. C.Z., P.L. and M.A. collected the samples from patients and contributed to data acquisition. M.A., A.Z., W.T. and B.F. conceived and designed the study and experiments. B.F., A.Z. and M.A. wrote and edited the paper. All authors read and approved the final manuscript.

## Abbreviations

circRNAs: circular RNAs
miRNAs: microRNAs
Pol II: RNA polymerase II
BMFS: brain metastasis-free survival
BRD4: bromodomain containing 4
MMP9: matrix metallopeptidase 9
SHH: Sonic hedgehog
BC: breast cancer
NBT: adjacent normal breast tissues
BCBM: breast cancer brain metastasis
ATCC: American Type Culture Collection
DMEM: dulbecco's modified eagle medium
FBS: fetal bovine serum
RT-qPCR: real-time quantitative PCR
FISH: fluorescence in situ hybridization
DAPI: 4,6-diamidino-2-phenylindole
MOI: multiplicity of infection
CCK8: cell counting kit-8
PFA: paraformaldehyde
H&E staining: hematoxylin and eosin staining
RIP: RNA immunoprecipitation
Ago2: Argonaute 2
PVDF: polyvinylidene fluoride
HRP: horseradish peroxidase
IHC: immunohistochemistry
SEM: standard error of the mean
CRC: colorectal cancer
BET: bromodomain and extra-terminal
