# Supplementary material for: Circular RNA circBCBM1 promotes breast cancer brain metastasis by modulating miR-125a/BRD4 axis
Source: Int J Biol Sci. 2021 Jul 22;17(12):3104–17. doi: 10.7150/ijbs.58916 (PMC8375234; doi:10.7150/ijbs.58916)
Supplement: Supplementary file 1 — Supplementary figures and tables. [file ijbsv17p3104s1.pdf]

Supplementary Table S1. Primer sequences.

Gene	Forward primer	Reverse primer
circBCBM1	GTCTGTTCCCAATACAAGAAGACTC	GAGCAGAGCAGCTTGAAGAACAT
Linear counterpartofcircBCBM1	GCGGTGACTCATGCCTGTAATCC	AGGCTGGTCTCGAACTCCTGAC
miR-125a*	GCGTCCCTGAGACCCTTTAAC	AGTGCAGGGTCCGAGGTATT
BRD4	CTCCCCGCTTATGATACATTCC	GTTTCTTAGGCTGGACGTTTTG
HDDC3	CACGAGGCGGGAATCACTGAC	ATCCAGGGTGGTGTCTGTGTCC
TXNRD1	ATATGGCAAGAAGGTGATGGTCC	GGGCTTGTCCTAACAAAGCTG

*Stem-loop Method; RT Primer: GTCGTATCCAGTGCAGGGTCCGAGGTATTCGCACTGGATACGACTCACAG

Supplementary Table S2. Bioinformatics analysis of the candidate miRNAs interacting with circBCBM1 using MiRanda Program.

miRNA	Total energy value	Strand	Length	Binding site
hsa-miR-125a-5p	-19.4	5408643	24	524
hsa-miR-1306-5p	-25.16	23867283	22	692
hsa-miR-34c-3p	-11.21	6847623	22	127
hsa-miR-26a-1-3p	-14.82	1017273	22	873
hsa-miR-10399-5p	-11.85	2839457	21	702
hsa-miR-661	-20.13	17143773	24	293

Supplementary Figure S1

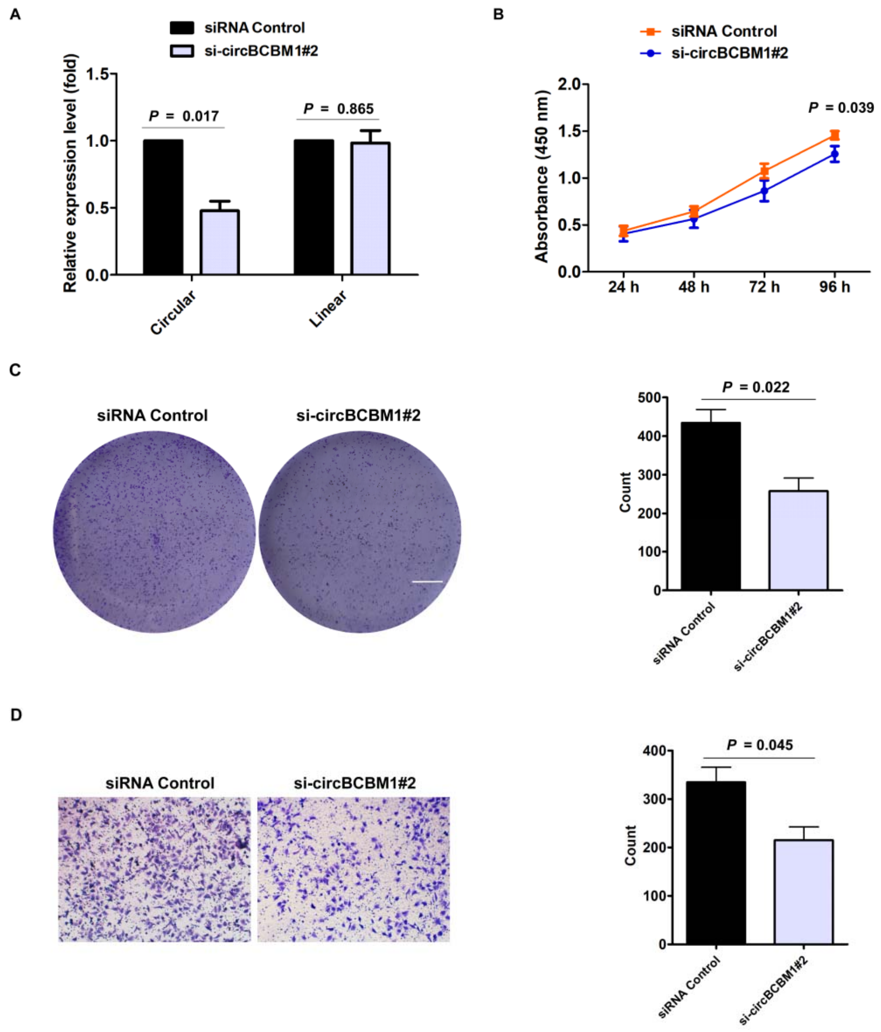

Supplementary Figure S1 Effects of si-circBCBM1#2 on cell proliferation, clone formation and migration capabilities. 231-BR cells were either transfected with si-circBCBM1#2 or siRNA Control. (A) RT-qPCR analysis of the expression level of circBCBM1 and its linear counterpart in 231-BR cells transfected with si-circBCBM1#2. The relative expression levels were normalized to the values in the siRNA-control group. (B) CCK-8 assay. (C) Colony formation assay. Scale bar, 5 mm. (D) Transwell migration assay. Data are presented as means ± SEM (A-D). si-circBCBM1#2, siRNA#2 against circBCBM1; siRNA Control, siRNA for negative control.

Supplementary Figure S2

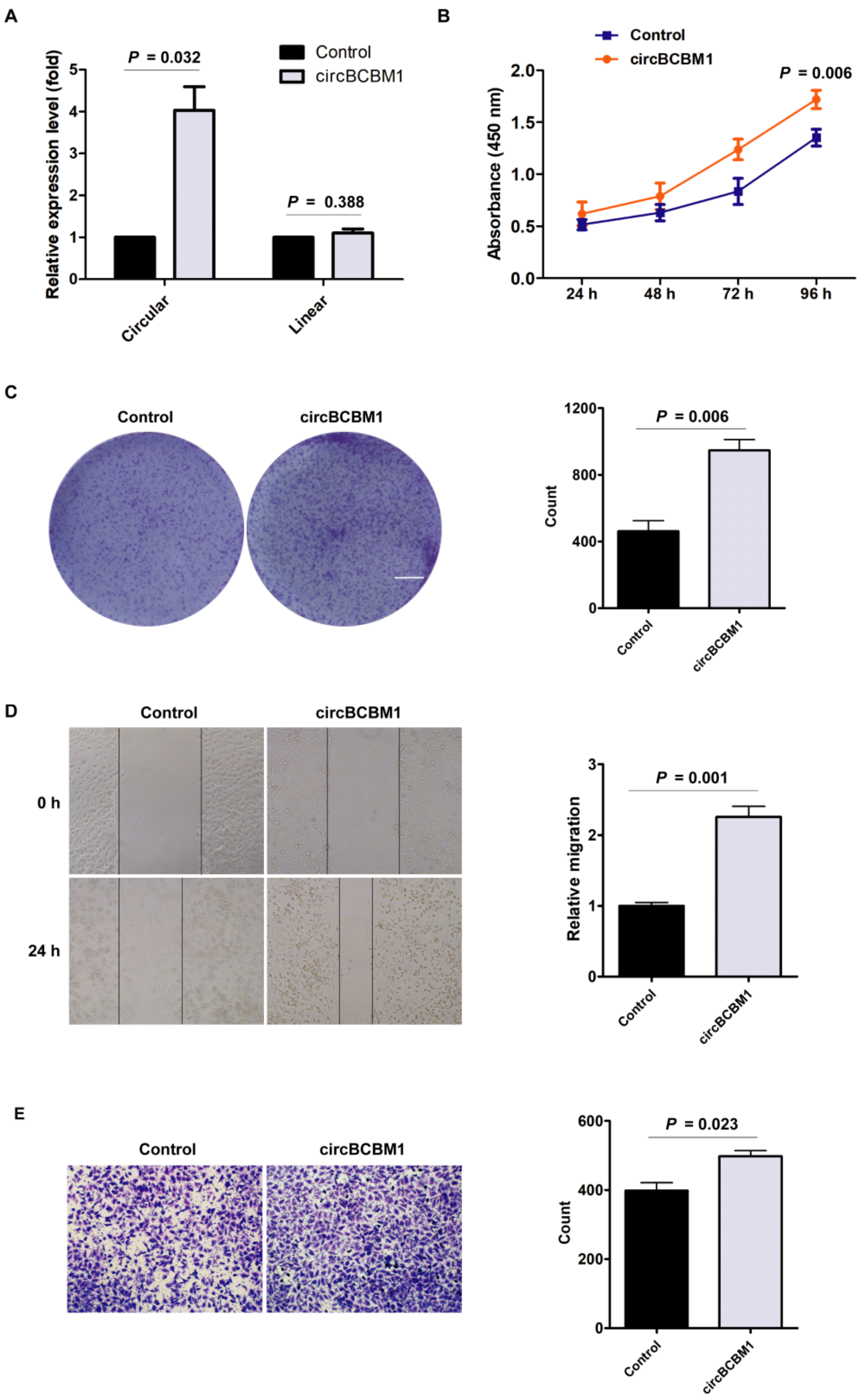

Supplementary Figure S2 Effects of circBCBM1 overexpression on the proliferation, clone formation and migration capabilities of 231-BR cells. (A) RT-qPCR analysis of circBCBM1 and its linear counterpart expression levels in 231-BR cells transfected with control vector (control group) or circBCBM1 over-expression plasmid (circBCBM1 group). The relative expression level was

normalized to that of the control group. (B) CCK-8 assay. (C) Colony formation assay. Scale bar, 5 mm. (D) Wound-healing assay. (E) Transwell migration assay. Data are presented as means ± SEM. Control group, 231-BR cells transfected with control vector; circBCBM1 group, 231-BR cells transfected with circBCBM1 over-expression plasmid.

Supplementary Figure S3

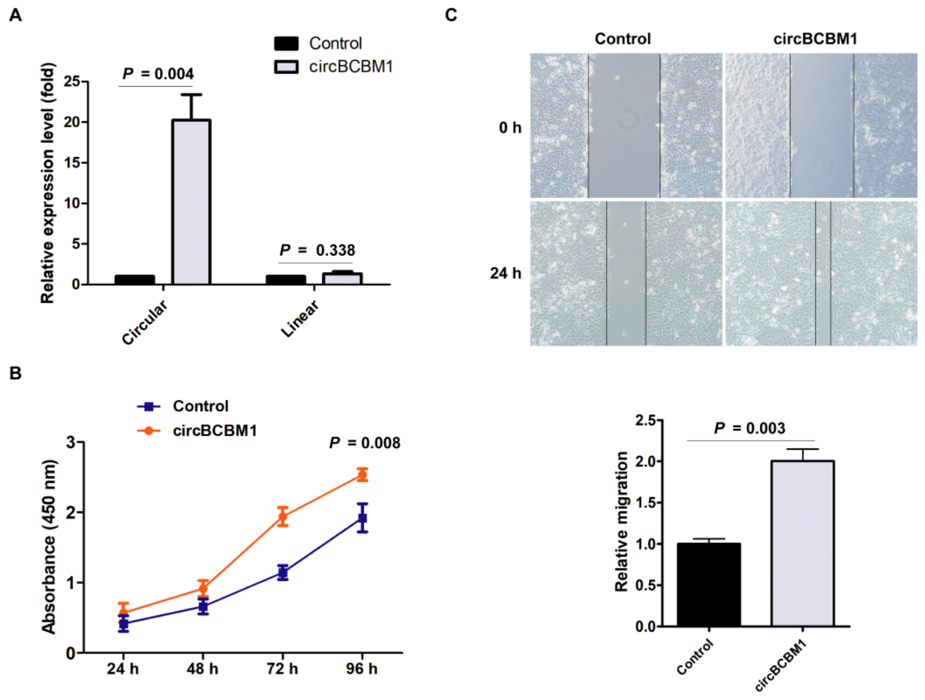

Supplementary Figure S3 Effects of circBCBM1 overexpression on the proliferation and migration capabilities of MDA-MB-231 cells. (A) RT-qPCR analysis of circBCBM1 and its linear counterpart expression levels in MDA-MB-231 cells transfected with control vector (control group) or circBCBM1 over-expression plasmid (circBCBM1 group). The relative expression level was normalized to that of the control group. (B) CCK-8 assay. (C) Wound-healing assay. Data are presented as means ± SEM. Control group, MDA-MB-231 cells transfected with control vector; circBCBM1 group, MDA-MB-231 cells transfected with circBCBM1 over-expression plasmid.

Supplementary Figure S4

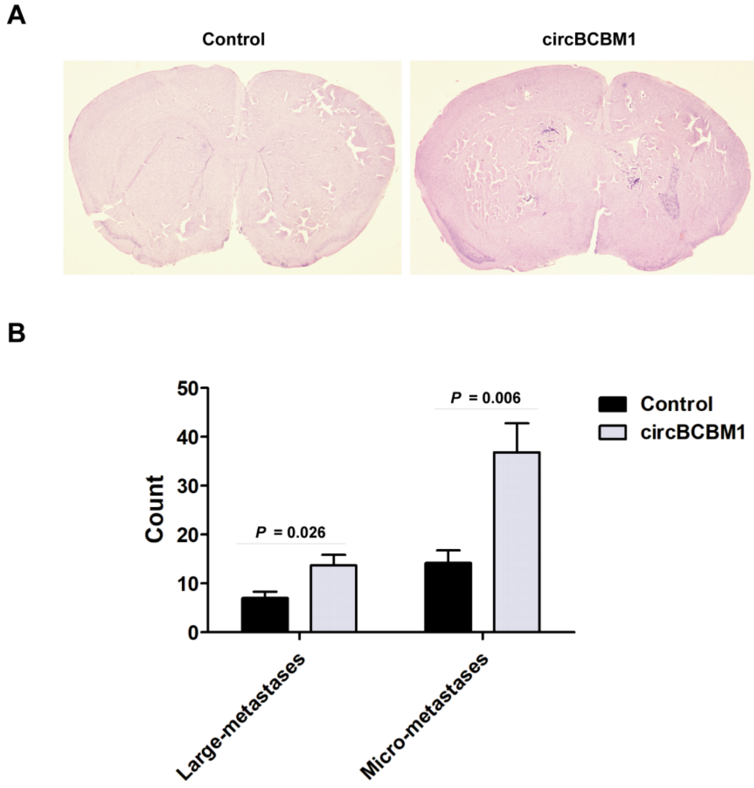

Supplementary Figure S4 Overexpression of circBCBM1 promotes brain metastasis of 231-BR cells *in vivo*. Brain metastasis models were generated by injection of 231-BR cells (1×10^5 per mouse), which were stably transfected with control lentivirus (control group) or circBCBM1 lentivirus (circBCBM1 group), into the left cardiac ventricle of mouse hearts. After 4 weeks, the brains were collected and metastatic nodules were counted after H&E staining. (A) Representative H&E staining images of brain metastases. (B) Quantification of the counts of the large- and micro-metastases. n = 6. Data are presented as means ± SEM.

Supplementary Figure S5

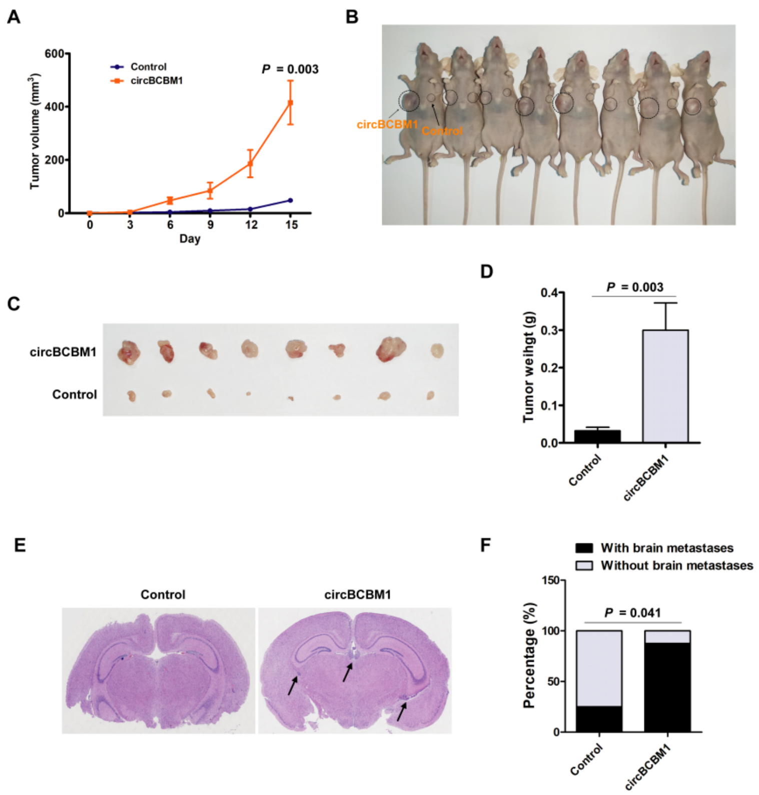

Supplementary Figure S5 Overexpression of circBCBM1 promotes growth and brain metastasis of MDA-MB-231 cells *in vivo*. Nude mice were subcutaneously injected MDA-MB-231 cells with stable transfection of control lentivirus (control group) or circBCBM1 lentivirus (circBCBM1 group) (n = 8) (A-D). (A) Tumor growth curves of subcutaneous models. (B) Images of the subcutaneous xenograft models on the 15th day. (C) Images of the dissected subcutaneous tumors. (D) Weights of the dissected subcutaneous tumors. Brain metastasis model was generated by injection of MDA-MB-231 cells (4×10^5 per mouse) with stable transfection of of control lentivirus (control group) or circBCBM1 lentivirus (circBCBM1 group) into the left cardiac ventricle of mouse heart. After 24 days, the brains were collected and then the metastasis status was detected by H&E staining (n = 8) (E and F). (E) Representative H&E images brain metastases. Arrows, metastasis nodules. (F) Percentage of mice with and without brain metastases. Data are presented as means ± SEM (A and D).

Supplementary Figure S6

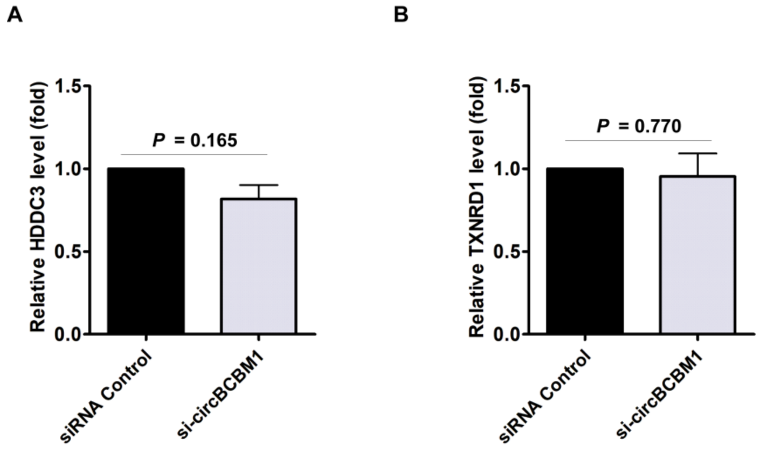

Supplementary Figure S6 RT-qPCR assays analyzed the expression levels of HDDC3 (A) and TXNRD1 (B) in 231-BR cells after treatment with si-circBCBM1. Data are presented as means ± SEM.

Supplementary Figure S7

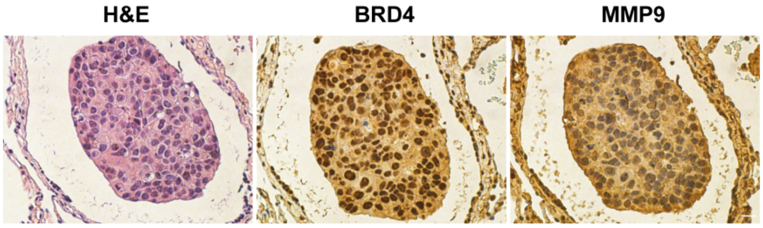

Supplementary Figure S7 Immunohistochemistry (IHC) staining of BRD4 and MMP9 in human breast cancer brain metastasis nodules. Left panel, H&E staining. Scale bar, 20 μm.
